# A machine learning-based prediction model pre-operatively for functional recovery after 1-year of hip fracture surgery in older people

**DOI:** 10.3389/fsurg.2023.1160085

**Published:** 2023-06-07

**Authors:** Chun Lin, Zhen Liang, Jianfeng Liu, Wei Sun

**Affiliations:** ^1^ Department of Orthopedics, Shenzhen Second People’s Hospital, the First Affiliated Hospital of Shenzhen University Health Science Center, Shenzhen, China; ^2^ Department of General Medicine and Geriatrics, Shenzhen Qianhai Shekou Free Trade Zone Hospital, Shenzhen, China; ^3^ Department of Geriatrics, Shenzhen People’s Hospital, Shenzhen, China; ^4^ Department of Cardiology, the Second Medical Center and National Clinical Research Center for Geriatric Diseases, Chinese People’s Liberation Army General Hospital, Beijing, China

**Keywords:** hip fracture surgery, machine learning, prediction model, functional recovery, older people

## Abstract

**Background:**

Machine learning (ML) has been widely utilized for constructing high-performance prediction models. This study aimed to develop a preoperative machine learning-based prediction model to identify functional recovery one year after hip fracture surgery.

**Methods:**

We collected data from 176 elderly hip fracture patients admitted to the Department of Orthopaedics and Oncology at Shenzhen Second People's Hospital between May 2019 and December 2019, who met the inclusion criteria. Patient's functional recovery was monitored for one year after surgery. We selected 26 factors, comprising 12 preoperative indicators, 8 surgical indicators, and 6 postoperative indicators. Eventually, 77 patients were included based on the exclusion criteria. Random allocation divided them into the training set (70%) and test set (30%) for internal validation. The Lasso method was employed to screen prognostic variables. We conducted comparisons among various common machine learning classifiers to determine the best prediction model. Prediction performance was evaluated using the area under the receiver operating characteristic curve (ROC), calibration curve, and decision curve analysis. To identify the importance of the predictor variables, we performed the recursive feature elimination (RFE) algorithm based on Shapley Additive Explanations (SHAP) values.

**Results:**

The AUCs for the testing dataset were as follows: logistic regression (Logit) model = 0.934, k-nearest neighbors (KNN) model = 0.930, support vector machine (SVM) model = 0.910, Gaussian naive Bayes (GNB) model = 0.926, decision tree (DT) model = 0.730, random forest (RF) model = 0.957, and Extreme Gradient Boosting (XGB) model = 0.902. Among the seven ML-based models tested, the RF model demonstrated the best prediction performance, incorporating four features: postoperative rehabilitation compliance, marital status, age-adjusted Charlson comorbidity score (aCCI), and clinical frailty scale (CFS).

**Conclusion:**

We developed a prediction model for the functional recovery following hip fracture surgery in elderly patients after one year, based on the Random Forest (RF) algorithm. This model exhibited superior prediction performance (ROC) compared to other models. The software application is available for use. External validation in a larger patient cohort or diverse hospital settings is necessary to assess the clinical utility of this tool.

## Introduction

The aging population is increasing in the contemporary world. By 2050, the United Nations (1) projects a population of 2.1 billion individuals aged 60 and above. Disabilities have a significant impact on the quality of life for older individuals, with hip fractures ranking among the most prevalent causes.

The international osteoporosis foundation (2, 3) reports that patients with hip fractures experience a one-year mortality rate of 20%–24%, with this heightened risk persisting for over 5 years (4, 5). Regarding functional prognosis, one year after hip fracture, about 40% of patients experience impaired independent ambulation, 60% require assistance, and 33% are fully reliant on daily support or reside in nursing homes (4, 6, 7). With an aging population, the prevalence of hip fractures in older individuals is projected to increase. By 2050, an estimated 6.3 million hip fractures will occur globally (8). Moreover, hip fractures impose a substantial economic burden on healthcare systems and society. The estimated cost of hip fractures in the United States was 17 billion dollars in 2015, and this figure is projected to reach 25.3 billion dollars by 2030 (9). The significant incidence, mortality, morbidity, and economic burden associated with hip fractures pose substantial social and medical challenges.

Predictive models for assessing the prognosis and potential complications of hip fractures are pivotal in understanding health outcomes related to this injury. These models employ diverse patient characteristics, clinical factors, and demographic data to predict outcomes, including mortality, functional recovery, and hospital readmission rates. An example of such a model is the Nottingham Hip Fracture Score (NHFS) (10), which is a well-established predictive model developed by the University of Nottingham. The NHFS was developed based on prospective collection of clinical and surgical data from 4,967 patients aged 65 years and older who underwent hip fracture surgery. The model includes six variables, namely age, pre-fracture residence, dementia status, presence of malignancy, serum creatinine level, and type of fracture, to predict 30-day mortality after hip fracture. Each variable is assigned a score, and the cumulative score is utilized to estimate the risk of mortality. The NHFS has demonstrated good discrimination and calibration in predicting mortality following hip fracture.

The use of ML techniques has facilitated the development of prognostic prediction tools in healthcare (11). These techniques enable the evaluation of real-world data that often exhibit complex nonlinear relationships. Moreover, machine learning can develop models with superior performance compared to traditional prediction methods (12), particularly for surgical procedures (13–15). Clinicians can enhance outcomes by developing high-performance prediction models that estimate the likelihood of favorable functional recovery. Given the alarming global population aging, the impact of physical and psychological changes associated with advanced age on the functional recovery of hip fracture surgery has garnered significant attention, particularly with respect to frailty and comorbidities. Currently, there is limited research on prognostic models for hip fractures in elderly patients considering age-related indicators. Consequently, this study seeks to develop a machine learning-based prediction model that incorporates age-related indicators to forecast the functional recovery of elderly patients one year after hip fracture surgery.

## Materials and methods

### Sources of data

We collected data from 176 hip fracture patients who were admitted to the Orthopaedics and Oncology Department of Shenzhen Second People's Hospital between May 2019 and December 2019 and met the inclusion criteria. The patients' functional recovery was assessed for one year following the surgery. Eventually, 77 patients were included based on the exclusion criteria. The research protocol was approved by the Clinical Research Ethics Committee of Shenzhen Second People's Hospital (approval number: 20200601052-FS01). Informed consent was obtained from each patient. The study was reported following the Transparent Reporting of a Multivariable Prediction Model for Individual Prognosis or Diagnosis (TRIPOD) statement (16).

### Study population

Based on the patient's medical history, symptoms, signs, and imaging examination, a diagnosis of either an intertrochanteric or femoral neck fracture was made.

Inclusion criteria: Patients aged 65 years or older, diagnosed with unilateral hip fracture (intertrochanteric or femoral neck fracture), and treated surgically at our hospital. The fractures were caused by low-energy injuries (fractures that occur in daily activities without significant external force or falls from a high altitude) and not high-energy injuries, such as car accidents. Patients had a complete medical history and provided informed consent for their condition and surgical treatment. Mental illness was an exclusion criterion if it hindered follow-up.

Exclusion criteria: Patients with pathological fractures (e.g., metastatic fractures). Fractures combined with other fractures in the same or opposite lower limb, disabilities, or other conditions leading to incomplete recovery of hip function were excluded. End-stage patients with severe postoperative complications and serious comorbidities who were unable to comply with the prescribed rehabilitation exercises were also excluded. Patients who were lost to follow-up, refused to participate in the follow-up, or died during the study were excluded.

### Data collection

The database was retrospectively reviewed to analyze the demographic and clinical characteristics of patients. These characteristics included 12 preoperative indicators: age, gender, marital status, BMI, polypharmacy, clinical frailty scale (CFS), age-adjusted Charlson comorbidity score (aCCI), fracture-admission time, preoperative waiting time, Morse fall scale, Barden score scale, and Caprini risk assessment for venous thromboembolism. Additionally, 8 surgical indicators were considered: fracture type, operation, anesthesia, ASA classification, postoperative white blood cell count, hemoglobin level, albumin level, and blood transfusion. Furthermore, 7 postoperative indicators were examined: length of stay, postoperative rehabilitation compliance, postoperative residence, postoperative caregiver, postoperative anticoagulant drugs, postoperative anti-osteoporosis drugs, and postoperative complications.

The data was reviewed by an investigator to ensure completeness, and the follow-up process was completed. To evaluate hip function recovery one year after the surgery, we utilized the Harris score (17) through telephone follow-up. The assessment content, consisting of 91 points, was evaluated through dialogue. Due to the COVID-19 pandemic, many older patients were unable to visit the hospital for evaluation. Therefore, this study recorded the 91 points that could be obtained via telephone follow-up. We then converted this score to a complete count, such as 91 points out of 100 points.

### Prediction model development

**Figure 1:**
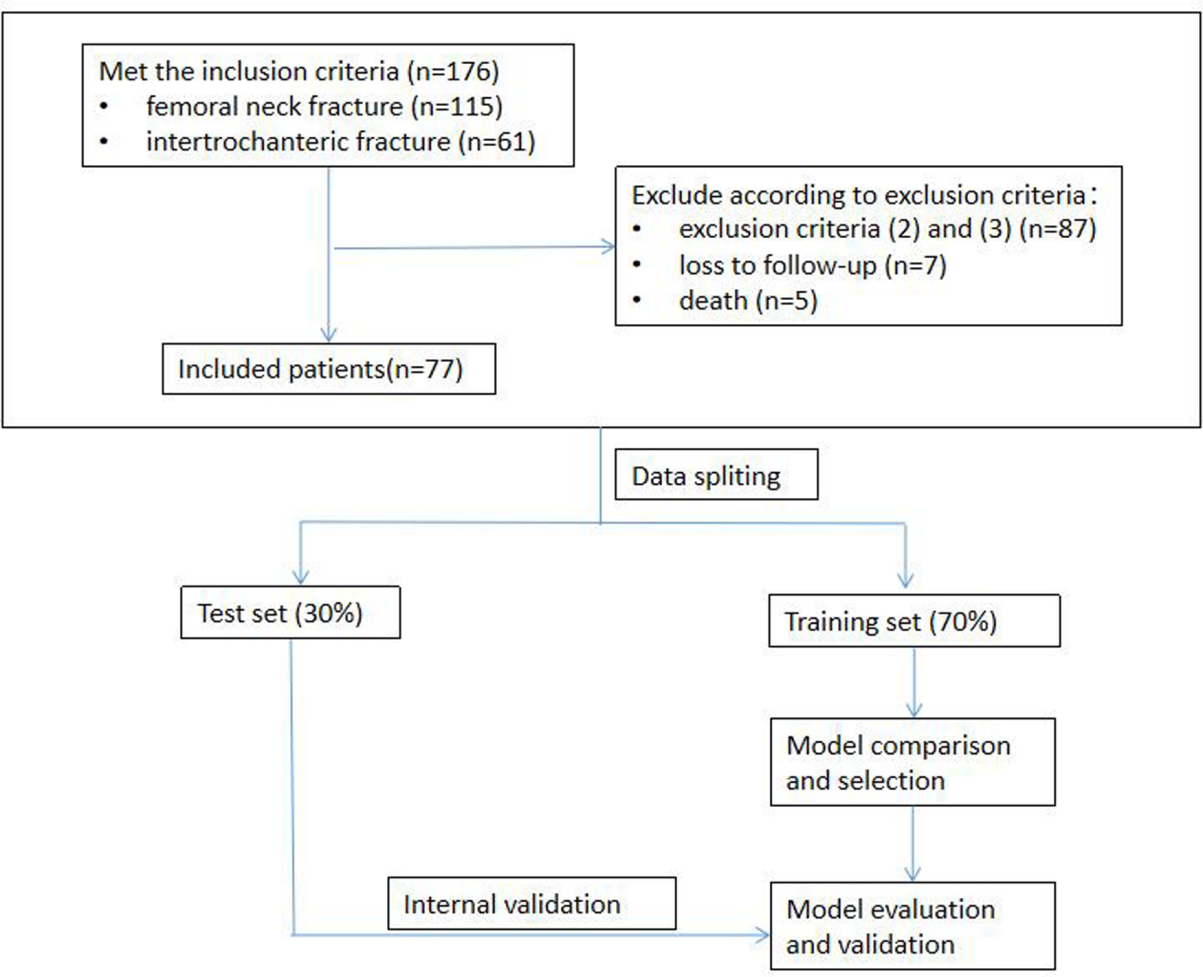
Data inclusion and exclusion flow and machine learning development process.

ML models were employed to develop a prediction model for the functional recovery of older individuals after 1-year hip fracture surgery. Initially, the Lasso regression was applied to screen pre-test variables in the prediction model. Subsequently, the database was randomly divided into a training set and a test set with a 7:3 ratio. We selected various standard ML classifiers, such as logistic regression (Logit), k-nearest neighbors (KNN), support vector machine (SVM), Gaussian naive Bayes (GNB), decision tree (DT), random forest (RF), and Extreme Gradient Boosting (XGB) models, to make initial predictions based on the variables identified through Lasso regression. Finally, the model with the highest AUC value was chosen as the final model (refer to Figure 1). To assess the importance of the feature variables, we utilized the recursive feature elimination algorithm based on SHAP values.

### Statistical analyses

Data normality was evaluated using the Shapiro-Wilk test. The i-test was utilized to compare continuous variables presented as mean ± standard deviation (SD). For non-normally distributed data, Mann–Whitney *U* tests were employed. Categorical data were compared using either a chi-squared test or Fisher's exact test. The model's discriminative ability in predicting functional recovery after 1-year of hip fracture surgery was assessed using the AUC. Detailed analysis of the model's performance involved calibration plots and decision curve analysis (DCA). Due to the small sample size, our study sample did not contain any missing values. Empowerstats version 4.0 (www.empowerstats.com) and Python version 3.7.0 (Python Software Foundation, www.python.org) were used for all statistical analyses. Statistical significance was determined using a two-tailed test with a significance level of *P* > 0.05.

## Results

### Baseline demographics and characteristics

**Figure 2:**
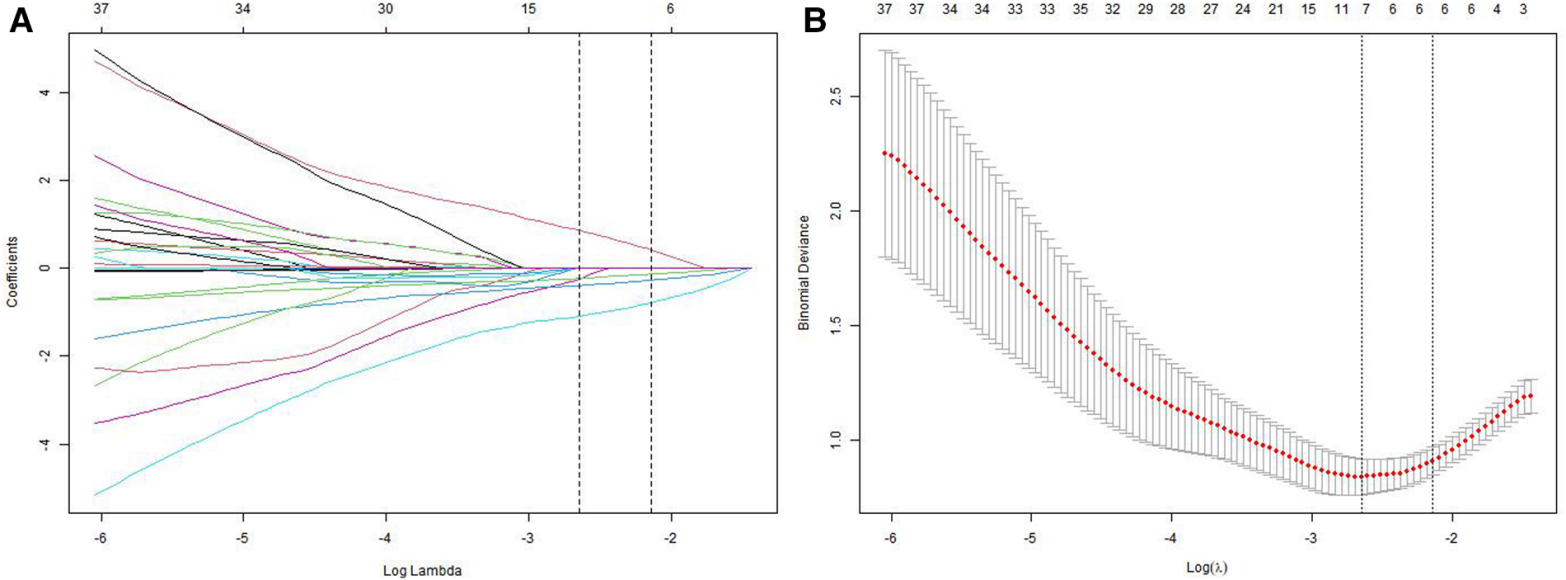
Lasso regression for screening prognostic variables. (A) A coefficient profile plot was generated based on the logarithmic sequence of lambda. (B) The binomial deviance curve was plotted against the logarithm of lambda in the Lasso model, and dotted vertical lines were added based on the 1 standard error criterion.

We analyzed a cohort of 77 older patients with hip fractures, of whom 21 experienced poor functional recovery. Furthermore, a comparison of 27 features, consisting of 12 preoperative indicators, 8 surgical indicators, and 7 postoperative indicators, was conducted to examine the demographic and characteristic differences between the poor functional recovery group (Harris score <70) and the good functional recovery group (Harris score ≥70) (refer to Tables 1, 2). Following Lasso regression (refer to Figure 2), we identified 4 crucial features, namely marital status, aCCI, CFS, and postoperative rehabilitation compliance, for developing a prediction model.

**Table 1:** Demographic and clinical characteristics of patients (preoperative and surgical indicators).

	Harris score		OR (95%CI)	*P*-value
	<70 (*n* = 21)	≥70 (*n* = 56)		
**Preoperative indicators**				
**Age**	86 ± 8	77 ± 7	1.13 (0.60, 1.66)	**<0**.**001**
Gender			0.38 (−0.13, 0.88)	0.177
Male	2 (9.52%)	13 (23.21%)		
Female	19 (90.48%)	43 (76.79%)		
**Marital status**			0.95 (0.43, 1.47)	**<0**.**001**
Married	11 (52.38%)	51 (91.07%)		
Widowed/divorced	10 (47.62%)	5 (8.93%)		
BMI	24.7 ± 9.1	22.5 ± 5.1	0.29 (−0.21, 0.80)	0.193
Polypharmacy	2 ± 2	1 ± 2	0.29 (−0.21, 0.80)	0.26
**aCCI**	6 ± 2	4 ± 1	1.17 (0.63, 1.70)	**<0**.**001**
**CFS**	5 ± 1	3 ± 1	1.44 (0.89, 1.99)	**<0**.**001**
Fracture-admission time(h)	40.8 ± 50.0	215.8 ± 634.1	0.39 (−0.12, 0.89)	0.212
Preoperative waiting time(h)	41.8 ± 31.3	40.1 ± 36.8	0.05 (−0.45, 0.55)	0.852
Morse fall scale	44 ± 9	40 ± 15	0.31 (−0.19, 0.82)	0.267
**Barden score scale**	13 ± 1	14 ± 1	0.67 (0.16, 1.18)	**0**.**019**
Caprini risk assessment for VTE	8.3 ± 1.8	8.1 ± 2.1	0.14 (−0.37, 0.64)	0.608
**Surgical indicators**				
Fracture type			0.38 (−0.13, 0.88)	0.139
Intertrochanteric fracture	11 (52.38%)	19 (33.93%)		
Femoral neck fracture	10 (47.62%)	37 (66.07%)		
**Operation**			0.95 (0.43, 1.48)	**0**.**016**
Artificial femoral head replacement	12 (57.14%)	15 (26.79%)		
Total hip replacement	2 (9.52%)	26 (46.43%)		
Internal fixation of intertrochanteric fracture	6 (28.57%)	14 (25.00%)		
Internal fixation of femoral neck fracture	1 (4.76%)	1 (1.79%)		
Anesthesia			0.46 (−0.05, 0.96)	0.246
Intraspinal anesthesia	17 (80.95%)	34 (60.71%)		
General anesthesia	3 (14.29%)	16 (28.57%)		
≥2 anesthesia types	1 (4.76%)	6 (10.71%)		
**ASA**			0.69 (0.18, 1.21)	**0**.**037**
2	3 (14.29%)	23 (41.07%)		
3	15 (71.43%)	31 (55.36%)		
4	3 (14.29%)	2 (3.57%)		
WBC	10.7 ± 5.9	10.9 ± 13.1	0.02 (−0.48, 0.52)	0.941
**Hg**	104.6 ± 21.3	116.2 ± 16.0	0.62 (0.10, 1.13)	**0**.**012**
**Albumin**	35.1 ± 6.3	39.8 ± 4.0	0.91 (0.26, 1.56)	**0**.**002**
**Transfusion**	3.6 ± 2.7	2.1 ± 2.0	0.63 (0.12, 1.14)	**0**.**009**

aCCI, age-adjusted Charlson comorbidity score; CFS, clinical frailty scale; ASA, American Society of Anesthesiologists; WBC, white blood cell; Hg, hemoglobin.

The bold *P*-value indicates that the value is statistically significant.

**Table 2:** Demographic and clinical characteristics of patients (postoperative indicators).

	Harris score		OR (95%CI)	*P*-value
	<70 (*n* = 21)	≥70 (*n* = 56)		
**Postoperative indicators**				
Length of stay	10.8 ± 3.3	10.3 ± 4.3	0.14 (−0.36, 0.64)	0.614
**Postoperative rehabilitation compliance**			1.34 (0.80, 1.89)	**<0**.**001**
Negative	15 (71.43%)	9 (16.07%)		
Positive	6 (28.57%)	47 (83.93%)		
Postoperative residence			0.34 (−0.16, 0.84)	0.118
Nursing home	2 (9.52%)	1 (1.79%)		
Home	19 (90.48%)	55 (98.21%)		
**Caregiver**			0.74 (0.23, 1.26)	**0**.**013**
Nursing workers	9 (42.86%)	7 (12.50%)		
Relatives	12 (57.14%)	48 (85.71%)		
Self Care	0 (0.00%)	1 (1.79%)		
Postoperative anticoagulant drugs			0.43 (−0.08, 0.93)	0.235
None	4 (19.05%)	6 (10.71%)		
Heparin	12 (57.14%)	43 (76.79%)		
Novel oral anticoagulants	5 (23.81%)	7 (12.50%)		
Postoperative anti-osteoporosis drugs			0.55 (0.04, 1.06)	0.057
None	6 (28.57%)	17 (30.36%)		
Single drug	5 (23.81%)	3 (5.36%)		
Two drugs	10 (47.62%)	36 (64.29%)		
Postoperative complications				
Anemia			0.14 (−0.36, 0.65)	0.571
No	7 (33.33%)	15 (26.79%)		
Yes	14 (66.67%)	41 (73.21%)		
Cardiac insufficiency			0.06 (−0.44, 0.56)	0.81
No	20 (95.24%)	54 (96.43%)		
Yes	1 (4.76%)	2 (3.57%)		
Hypoalbuminemia			0.42 (−0.09, 0.92)	0.113
No	7 (33.33%)	30 (53.57%)		
Yes	14 (66.67%)	26 (46.43%)		
**Electrolyte disturbance**			0.54 (0.03, 1.05)	**0**.**018**
No	16 (76.19%)	53 (94.64%)		
Yes	5 (23.81%)	3 (5.36%)		
Pneumonia			0.30 (−0.20, 0.81)	0.193
No	18 (85.71%)	53 (94.64%)		
Yes	3 (14.29%)	3 (5.36%)		
Phlebothrombosis			0.10 (−0.40, 0.60)	0.706
No	20 (95.24%)	52 (92.86%)		
Yes	1 (4.76%)	4 (7.14%)		
Urinary infection			0.03 (−0.47, 0.53)	0.917
No	20 (95.24%)	53 (94.64%)		
Yes	1 (4.76%)	3 (5.36%)		
Pulmonary embolism			0.19 (−0.31, 0.69)	0.538
No	21 (100.00%)	55 (98.21%)		
Yes	0 (0.00%)	1 (1.79%)		
Hepatic dysfunction			0.17 (−0.33, 0.67)	0.493
No	18 (85.71%)	51 (91.07%)		
Yes	3 (14.29%)	5 (8.93%)		
Delirium			0.19 (−0.31, 0.69)	0.538
No	21 (100.00%)	55 (98.21%)		
Yes	0 (0.00%)	1 (1.79%)		

The bold *P*-value indicates that the value is statistically significant.

### RF model development

**Figure 3:**
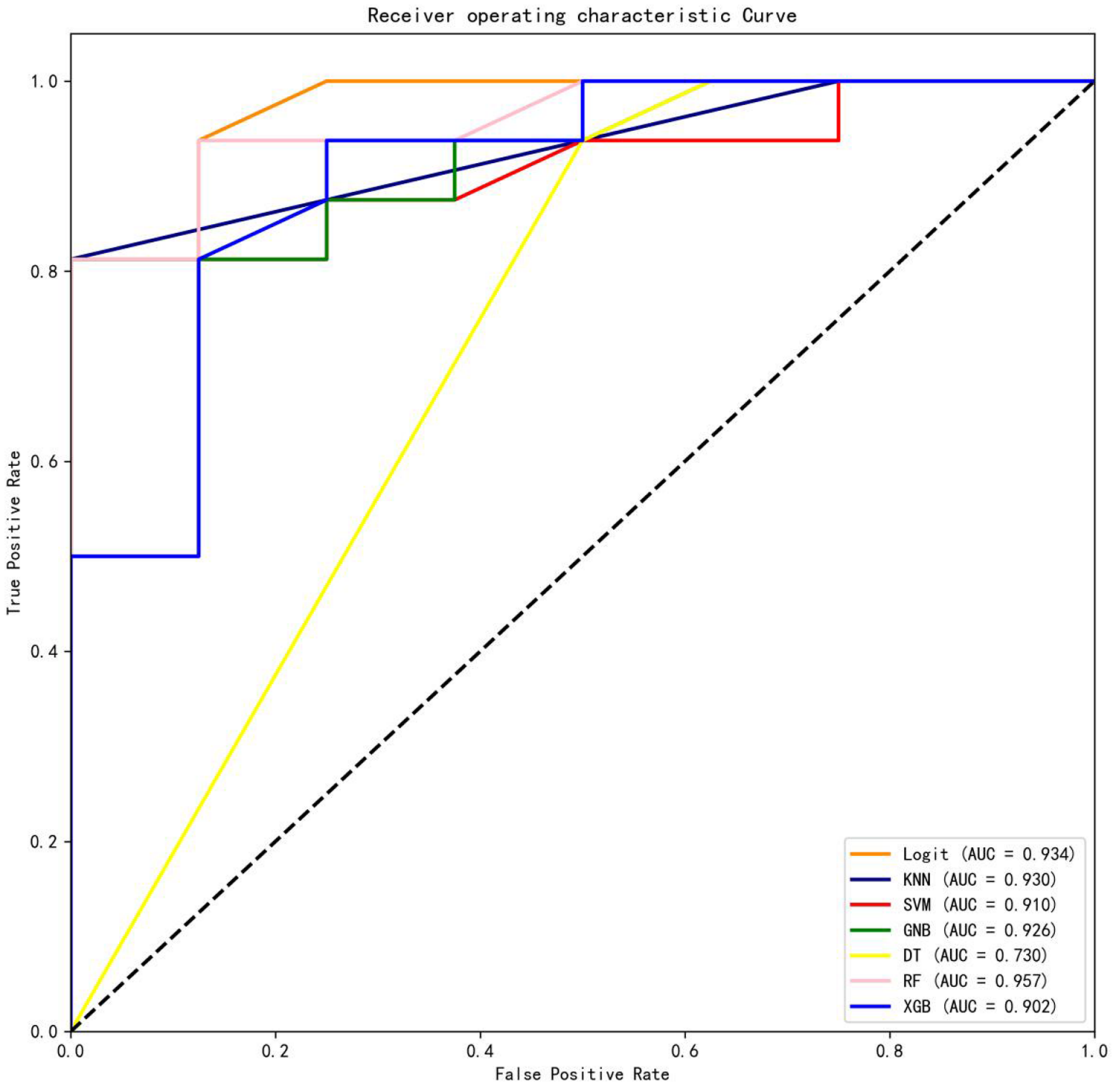
Comparisons of different machine learning models. Logit, logistic regression; KNN, k nearest neighbors; SVM, support vector machine; GNB, Gaussian naive Bayes; DT, decision tree; RF, random forest; XGB, Extreme Gradient Boosting.

**Figure 4:**
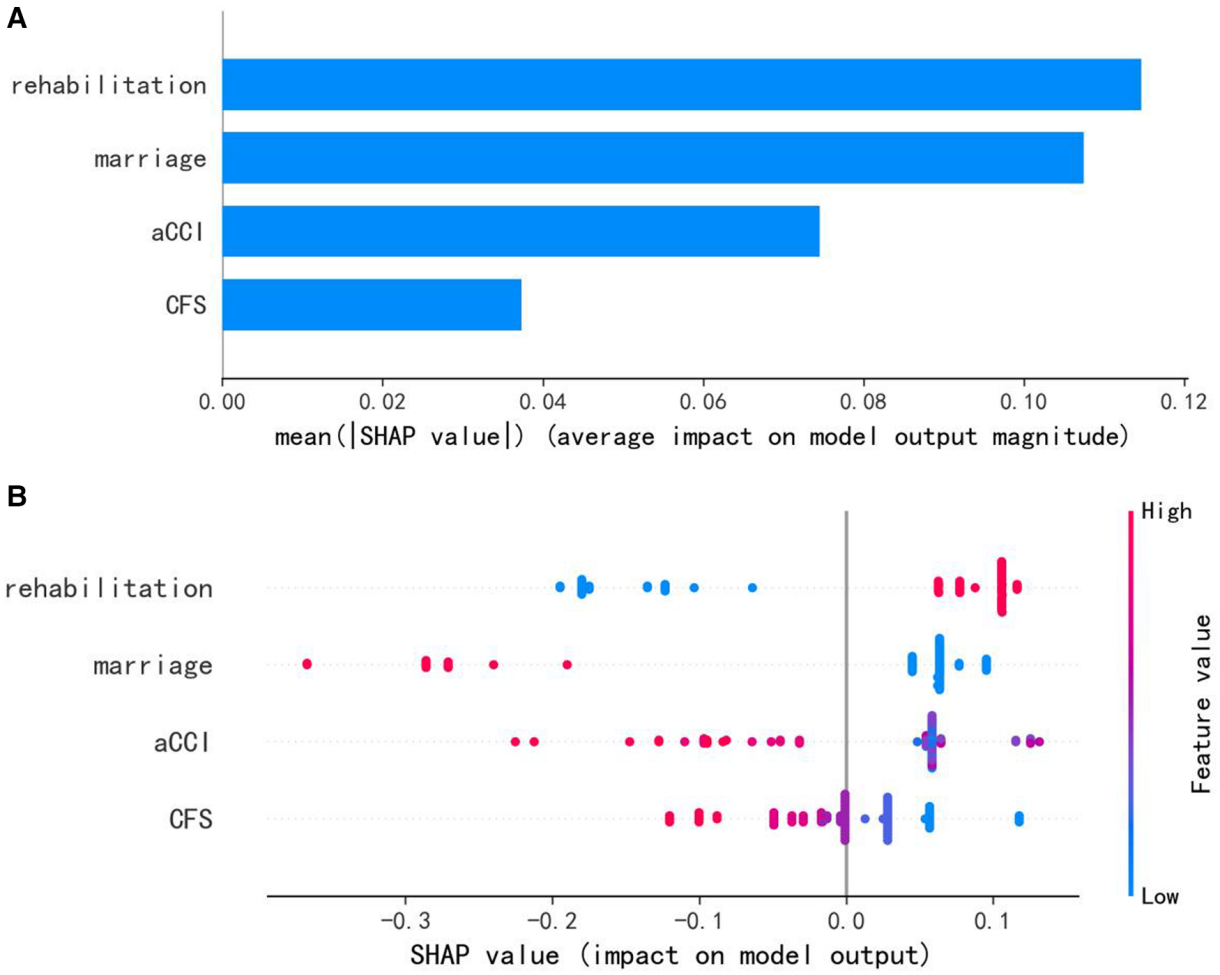
(**A**) characteristics of the selected model (random forests model): SHAP value summary graph of variables and their impact on the prediction. (**B**) Importance of the predictor variables in the random forest model. aCCI, age-adjusted Charlson comorbidity score; CFS, clinical frailty scale.

Among the seven machine learning-based models, the RF algorithm exhibited the highest prediction performance (AUC = 0.957) for the initial prediction of functional recovery (refer to Figure 3). Consequently, the RF model was chosen as the ultimate prediction model. The impact of each feature on the final model results is depicted in Figure 4A, while Figure 4B presents the assessment of feature importance using SHAP values.

### Model evaluation and validation

**Figure 5:**
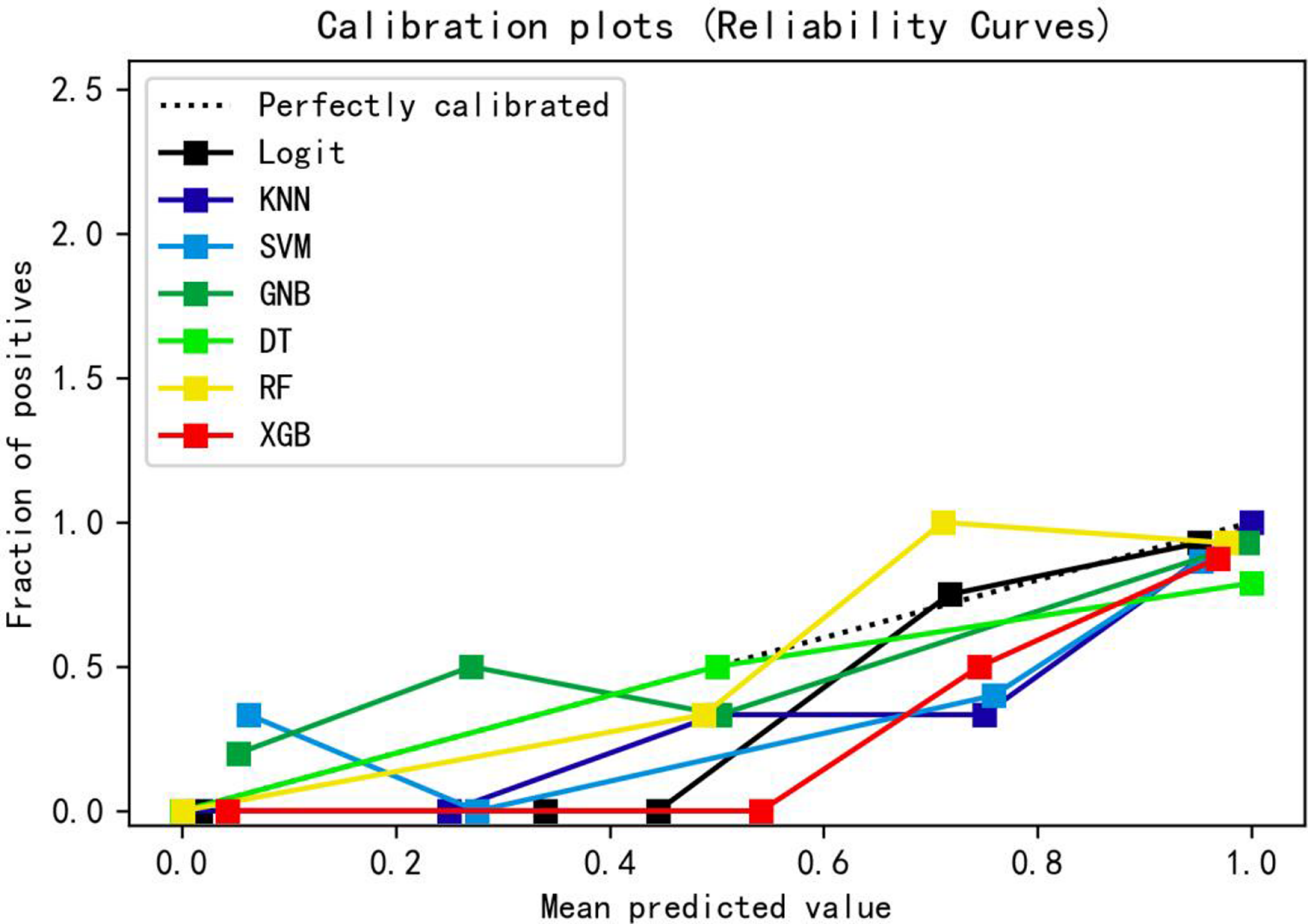
Calibration curve of different machine learning models. Logit, logistic regression; KNN, k nearest neighbors; SVM, support vector machine; GNB, Gaussian naive Bayes; DT, decision tree; RF, random forest; XGB, Extreme Gradient Boosting.

**Figure 6:**
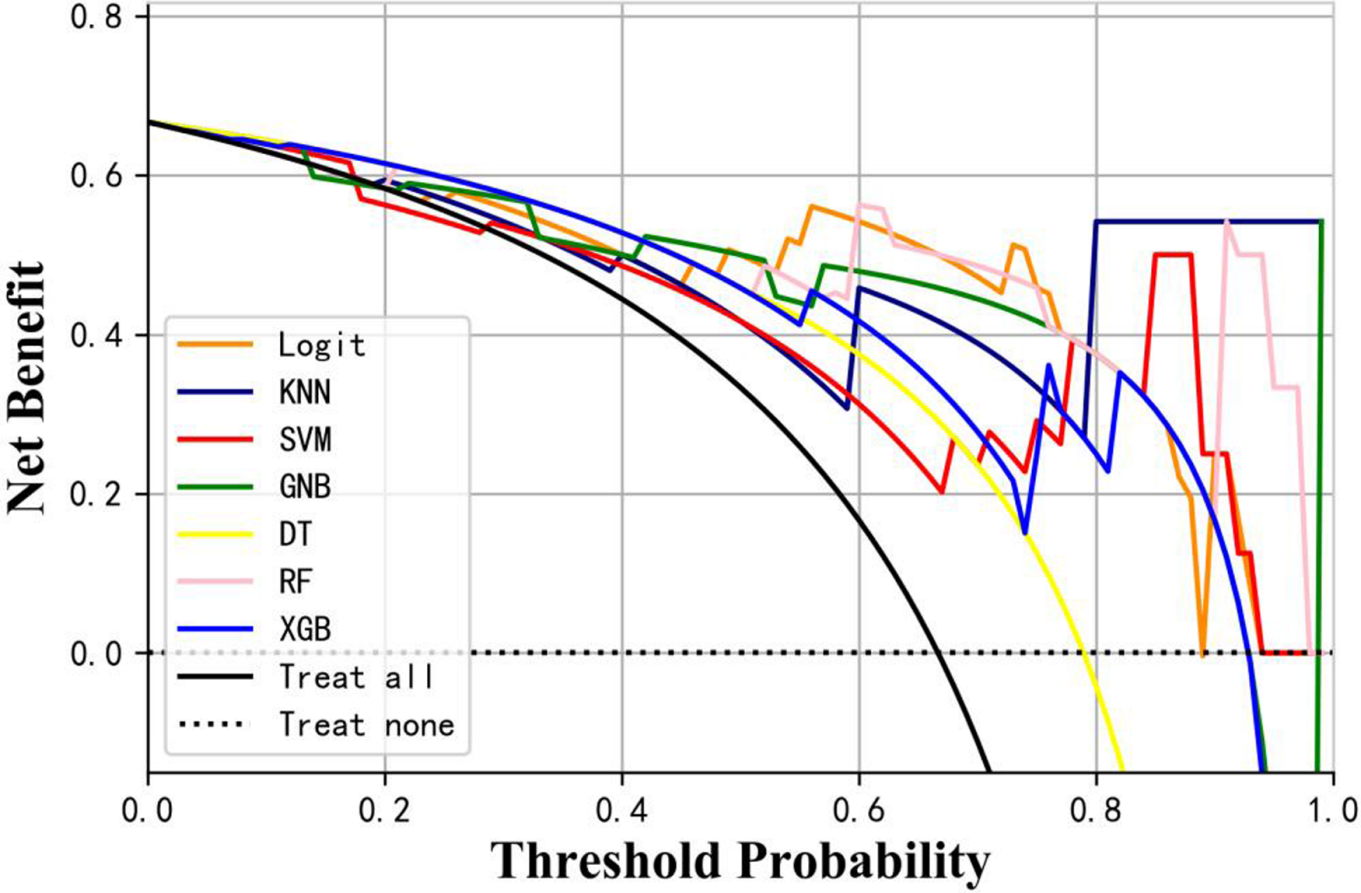
Decision curve analysis of different machine learning models. Logit, logistic regression; KNN, k nearest neighbors; SVM, support vector machine; GNB, Gaussian naive Bayes; DT, decision tree; RF, random forest; XGB, Extreme Gradient Boosting.

**Figure 7:**
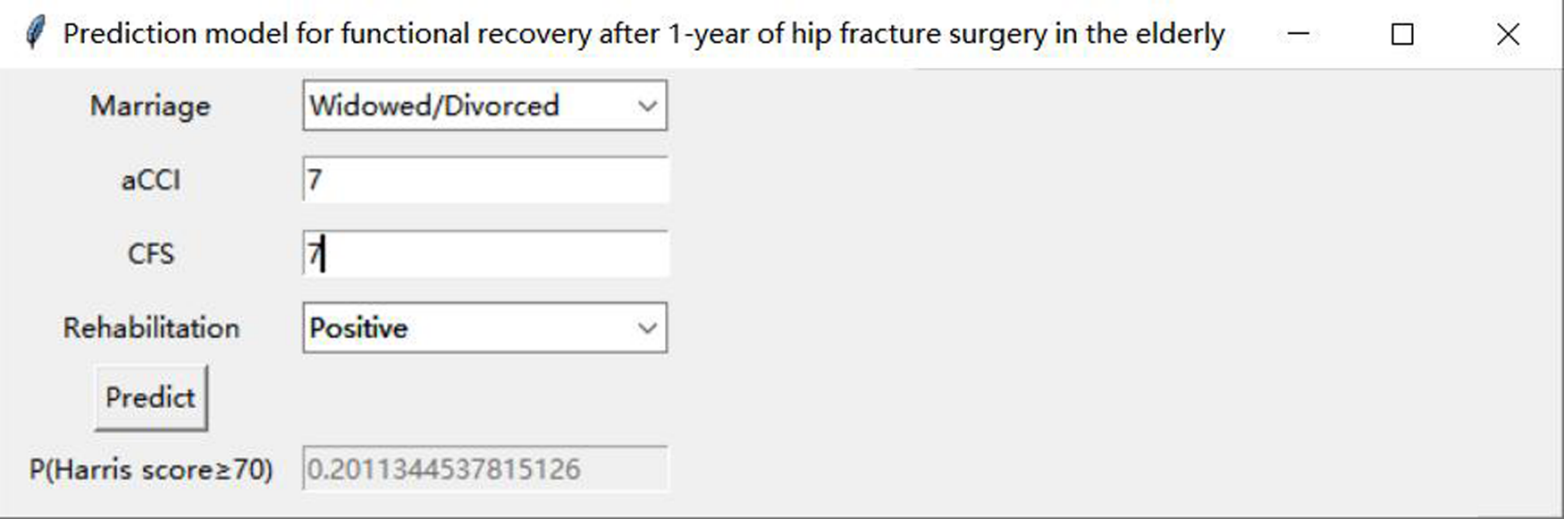
An example of the prediction software. aCCI, age-adjusted Charlson comorbidity score; CFS, clinical frailty scale.

Table 3 presents the evaluation indicators, including AUC, accuracy, sensitivity, specificity, positive predictive value (PPV), negative predictive value (NPV), F1-score, and Matthews correlation coefficient (MCC), for each model using the 4 features in the internal validation sets. The RF model achieved the highest values in AUC, specificity, and PPV, as indicated in Table 3. Furthermore, calibration curve plotting and decision curve analysis (DCA) were performed in this study. To simplify the comparison, the Logit, KNN, SVM, GNB, DT, and XGB models were compared with the RF model (refer to Figures 5, 6). Finally, a software program was developed based on the 4 features to predict the probability of good functional recovery (refer to Figure 7). The predictive software of this study is available on Baidu Netdisk (https://pan.baidu.com/s/16Iq93rxvu8fh5PTgYsqZNw?pwd=ea6z, code: ea6z).

**Table 3:** Model evaluation.

Model_name	AUC	Accuracy	Sensitivity	Specificity	PPV	NPV	F1	MCC
Logit	0.934	0.917	0.938	0.875	0.938	0.875	0.938	0.813
KNN	0.930	0.875	0.813	1.000	1.000	0.727	0.897	0.769
SVM	0.910	0.875	0.813	1.000	1.000	0.727	0.897	0.769
GNB	0.926	0.875	0.813	1.000	1.000	0.727	0.897	0.769
DT	0.730	0.792	0.938	0.500	0.789	0.800	0.857	0.508
RF	0.957	0.875	0.813	1.000	1.000	0.727	0.897	0.769
XGB	0.902	0.833	0.813	0.875	0.929	0.700	0.867	0.657

Logit, logistic regression; KNN, k nearest neighbors; SVM, support vector machine; GNB, Gaussian naive Bayes; DT, decision tree; RF, random forest; XGB, Extreme Gradient Boosting; PPV, positive predictive value; NPV, negative predictive value; MCC, Matthews correlation coefficient.

## Discussion

This study developed a prediction model using machine learning (ML) algorithms. Among the 7 ML models tested in the internal validation set, the random forest (RF) model demonstrated the highest performance. The RF model revealed that being widowed or divorced, having high scores of age-adjusted Charlson comorbidity index (aCCI) and clinical frailty scale (CFS), and exhibiting negative rehabilitation compliance were significant risk factors for functional recovery. Conversely, being married, having low scores of aCCI and CFS, and exhibiting positive rehabilitation were significant protective factors for functional recovery. These findings indicate that our prediction model can identify patients at risk of poor functional recovery prior to surgery, enabling early intervention. The initial step involves effective communication with patients and their families regarding treatment options and associated risks. To ensure optimal utilization of multidisciplinary resources, healthcare providers, families, and caregivers should promptly make clinical decisions, including regular monitoring and optimization of patients' medical conditions, increased home visits, caregiver support, and tailored rehabilitation programs.

### Logistic regression (LR)

Logistic regression (LR) is a generalized linear regression analysis model that employs the sigmoid function to accurately map the regression value to the range of 0–1, enabling precise prediction of an event's probability (18). LR finds wide application in numerous fields, including the automated diagnosis of diseases, due to its remarkable interpretability, prediction accuracy, and computational efficiency. However, the model tends to underfit when dealing with complex feature spaces, requiring users to manually construct features in order to enhance model accuracy.

### k nearest neighbors (KNN)

The k nearest neighbors (KNN) is a non-parametric learning algorithm that determines the category of a sample by conducting a vote among its k nearest training samples (19). Typically, the Euclidean distance is employed to measure the distance between samples. KNN is applicable for nonlinear classification without making assumptions about data distribution. However, its accuracy tends to suffer in the presence of imbalanced samples.

### Support vector machine (SVM)

The support vector machine (SVM) employs a hyperplane to classify samples into different categories within the input space (20). The learning process involves finding the coefficients of the hyperplane that optimally separates the classes. The model is capable of handling high-dimensional feature problems and capturing nonlinear characteristics. However, finding an appropriate kernel function can be challenging, and SVM is typically used for solving binary classification problems.

### Gaussian naive Bayes (GNB)

The Gaussian naive Bayes (GNB) (21) is based on the Bayesian theorem and the naive hypothesis, assuming that each feature within each category follows a normal distribution. Probabilities are calculated using the probability density function of the normal distribution for each feature in each category. The model exhibits a fast prediction speed and is applicable to multi-classification problems; however, its effectiveness heavily relies on the underlying data distribution.

### Decision tree (DT)

The decision tree (DT) is employed as a tree-based model for solving classification problems (22). Nodes represent the decision conditions, while the leaves indicate the decision outcomes. The objective of optimization is to minimize information entropy. The DT model is characterized by its high interpretability and can be transformed into decision rules using if-else-then statements. Nevertheless, the conventional decision tree algorithm tends to overfit easily and disregards feature interactions.

### Extreme gradient boosting (XGB)

The Extreme Gradient Boosting (XGB) is an ensemble of classification and regression trees trained using a boosting technique called integrated learning (23). The XGB model is capable of handling both classification and regression problems. It can achieve high prediction accuracy even without extensive parameter tuning, outperforming many other machine learning models. However, the model is not suitable for processing unstructured data types, such as images and texts.

### Random forest (RF)

The random forest (RF) is an ensemble learning algorithm that constructs multiple decision trees in random subspaces of the feature space, which helps preserve the generalization capability compared to traditional training methods for decision trees (24). Several studies have demonstrated the reliable classification capability of RF in medical applications (25, 26). Compared to other models, the RF algorithm exhibited superior prediction results in this study, demonstrating its exceptional generalization capability. To develop robust prediction models, we recommend utilizing the RF algorithm.

While it is true that most machine learning algorithms are capable of handling complex relationships between variables and outcomes, interpreting real-world problems can present certain challenges (27). To comprehend the contribution of each predictor variable to the outcome, one can rank the variables based on their impact on the model using SHAP values (27, 28). Figures 4, 5 depict SHAP-value graphs that illustrate the influence of variables on predictions made by the RF model.

Undoubtedly, rehabilitation plays a pivotal role in facilitating postoperative functional recovery. Several high-quality studies (29, 30) have provided evidence that postoperative rehabilitation can enhance hip function following hip fracture surgery. Furthermore, Ziden et al. (29) reported that home-based physical therapy yielded positive outcomes in terms of physical functioning, activities of daily living, and patient satisfaction. High-quality studies (31, 32) provide additional evidence that intensive exercise training combined with physical therapy after discharge leads to improved functional outcomes. A study (31) demonstrated that home-based leg-strengthening exercises significantly enhanced leg muscle strength, gait speed, and the 6-minute walk distance among community-dwelling older patients recovering from hip fracture over a 6-month period. In a randomized controlled trial involving a 3-month leg-muscle strength training program (32), rehabilitation was found to enhance mobility and instrumental activities of daily living in patients recovering from hip fracture surgery while residing at home.

This finding aligns with our prediction model, confirming that rehabilitation is the primary determinant influencing postoperative functional recovery. Moreover, we hypothesize that marital status, the variable with the second highest weight in our prediction model, indirectly influences rehabilitation and, consequently, postoperative functional recovery. In contrast to married patients with surviving spouses, widowed or single patients may engage in reduced outdoor activities, limited social interactions, decreased exercise, and exhibit poor adherence to rehabilitation due to the absence of a companion. These factors can impact the recovery of hip function following surgery. In a qualitative study (33), it was found that the involvement of caregivers plays a crucial role in providing physical and emotional care for patients with hip fractures. In a literature review conducted by Rocha et al. (34), it was found that older patients with hip fractures are typically attended to by female family members following surgery. In the majority of cases, the spouse assumes the role of the primary caregiver, and informal caregivers play a beneficial role in promoting the postoperative functional independence of patients.

The coexistence of multiple diseases is characteristic of older individuals. The Age-adjusted charlson comorbidity index (aCCl) (35) is a widely utilized scoring system for comorbidities that accounts for age factors in addition to the CCI. Typically, it is employed to estimate mortality rates attributed to comorbidities, considering their number and severity. According to a retrospective analysis (36), there were no significant differences in functional outcomes after surgery at 1 or 2 weeks regarding CCI. These findings suggest that CCI may not have a statistically significant impact on short-term functional recovery. Our study revealed a significant increase in aCCI among the poor recovery group, indicating that aCCI serves as a predictor of functional recovery one year after hip fracture surgery.

Frailty is a prevalent geriatric syndrome. However, there is a lack of consistent criteria for assessing and diagnosing frailty. Various frailty assessment methods exist, including the Fried frailty phenotype, FRAIL scale SOF Index, and CFS (37). Kistler et al. (38), using the Fried frailty phenotype, identified frailty in up to 51% of older patients with hip fractures. However, conducting frailty assessments among surgeons poses challenges due to varying assessment criteria, multiple assessment items, complex assessment content, and the requirement for specialized assessment equipment. Through a literature review, Dent et al. (37) found that the CFS had a short evaluation time, averaging less than 5 min. A detailed, comprehensive geriatric assessment was unnecessary, as the degree of frailty could be assessed and graded solely based on charts and descriptions, making it suitable for rapid assessment in clinical practice. Chen CL et al. (39), defining frailty using the CFS, observed that hip fracture surgery patients over the age of 50 with frailty experienced a poor prognosis and high mortality rate. These findings align with our conclusion that CFS values were higher in the poor recovery group, indicating its predictive role in the functional recovery of older individuals one year after fracture surgery.

Our machine learning (ML)-based prediction model aids clinicians in making clinical decisions by accurately predicting patients who are likely to experience poor postoperative functional recovery through preoperative analysis. Additionally, it can serve as a motivator for physicians to provide counseling to patients and caregivers, thereby promoting adherence to medical interventions.

### Strengths and limitations

Our study resulted in the development of a predictive model for assessing the functional recovery of older patients undergoing hip fracture surgery after one year. To the best of our knowledge, no machine learning (ML)-based prediction model has been developed to assess the functional recovery following one-year hip fracture surgery in older patients. Moreover, we evaluated several machine learning (ML) algorithms to determine their accuracy in prediction. Additionally, we developed an online software program capable of predicting the probability of favorable functional recovery.

The present study had several limitations. Firstly, the Harris score was evaluated and recorded only through telephone follow-up, resulting in a maximum score of 91 out of 100 points. Therefore, we adjusted the score to reflect the complete range. For instance, a score of 91 points was adjusted to 100 points. Secondly, the prediction model underwent internal validation without external validation. While internal validation indicated favorable predictive ability, external validation is necessary for further validation. Thirdly, due to the small sample size, our study did not include a comparison of patient demographics and characteristics between the training set and test set.

## Conclusion

A prediction model based on ML algorithms was developed, incorporating four features: marital status, aCCI, CFS, and postoperative rehabilitation compliance. The RF model demonstrated the highest AUC and specificity among the seven models tested, enabling preoperative prediction of functional recovery after one year of hip fracture surgery in older patients. Additionally, the software for this model is accessible online. Future external validation is necessary to validate these findings.

## Data Availability

The raw data supporting the conclusions of this article will be made available by the authors, without undue reservation.
